# The Evolutionary Psychology of Breaking Informal Versus Formal Contracts: Effects of Group Size and Area of Upbringing

**DOI:** 10.3390/bs15111458

**Published:** 2025-10-26

**Authors:** Glenn Geher, Ethan Eisenberg, Michael DeMaio, Olivia Casa, Anthony J. Caserta, Katherine Cochran, Leah Cohen, Aliza Dewan, Stephanie Dickinson-Frevola, Lauryn Fenigstein, Chloe Giboyeaux, Mia Goren, Emma Jerabek, Julia Lieberstein, Lindsay Marr, Brandon Staccio, Nadia Tamayo

**Affiliations:** 1 Department of Psychology, State University of New York at New Paltz, New Paltz, NY 12561, USA; geherg@newpaltz.edu (G.G.); casertaa1@newpaltz.edu (A.J.C.); dickinss2@newpaltz.edu (S.D.-F.); jerabeke1@newpaltz.edu (E.J.); stacciob1@newpaltz.edu (B.S.); tamayon1@newpaltz.edu (N.T.); 2 Doctoral Program in School-Community Psychology, Hofstra University, Hempstead, NY 11550, USA

**Keywords:** social contract, cheater detection, evolutionary mismatch, evolutionary psychology, Environment of Evolutionary Adaptedness

## Abstract

The social context for human social interactions between modern urban contexts and ancestral, small-scale contexts is different in many important ways. Before the advent of agriculture, all people lived in small-scale social contexts and were surrounded by kin and other familiar others. As these conditions characterized the lion’s share of human evolutionary history, we can expect much of our social psychology to be more designed for such small-scale contexts than for large-scale contexts. The study described here specifically predicted that informal forms of making an agreement (such as a handshake, which is more similar to how contracts are sealed in small-scale societies) would be weighted more heavily by people who are given an option to break a contract in a small-scale context. On the other hand, we predicted that people who are framed to think about large-scale social contexts will give more weight to written contracts. Using a 2*2 between-groups design (with 200 young adult participants), this interaction-based hypothesis was supported. We also found that, apart from experimental conditions, participants who reported coming from urban backgrounds were more likely to break a deal of any kind relative to others. Implications for cultivating prosocial outcomes against this backdrop are discussed.

## 1. The Evolutionary Psychology of Breaking Informal Versus Formal Contracts: Effects of Group Size and Area of Upbringing

During the lion’s share of human evolutionary history, people tended to live in small groups constituted largely by kin as well as other familiar individuals (see Geher & Wedberg, 2022). Further, during these critical years comprising the human Environment of Evolutionary Adaptedness (EEA), the only form of communication was, by and large, of the face-to-face variety. An implication of these points is that people evolved to (a) interact primarily with familiar others (b) in face-to-face scenarios.

These days, we tend to see large-scale evolutionary mismatch when it comes to our social world (see Giphart & Van Vugt, 2018). Evolutionary mismatch essentially pertains to situations in which the current context of some organism is, in important ways, mismatched from the ancestral conditions that surrounded the organism’s ancestors. Under ancestral conditions, before the advent of agriculture, all people were nomads living in small-scale social contexts. Urban living, characterized by large numbers of people who are often strangers to one another, living together, is quite evolutionarily novel (and, thus, leads to a relatively large case of evolutionary mismatch).

In terms of the points at hand, in many industrialized contexts that surround so many modern humans, people often find themselves in large cities communicating with strangers—often in a non-face-to-face format.

Many predictions can be made regarding the fact that modern social environments are evolutionarily mismatched from the small-scale social ecology of the human EEA. One outcome associated with living in the modern industrialized world is the fact that we so-often interact with people whom we would expect to never see again in our lives (this all has to do with the psychology of dealing with strangers, which is famously different from the psychology of dealing with familiar others and kin members; see Geher et al., 2023). In short, people tend to be more likely to behave in anti-social, as opposed to prosocial, ways in dealing with strangers whom they may never see again (see Zimbardo, 2007).

Similarly, given the fact that people are now easily capable of communicating with one another in non-face-to-face ways—often anonymously—we can predict that such a communication–based mismatch may lead to increased levels in anti-social behaviors (cf., Geher et al., 2023).

So there are a few different ways that our modern, post-industrialized contexts differ from the social ecosystems that surrounded most of our evolutionary history. For one, there were no large-scale societies under ancestral, nomadic conditions (see Dunbar, 1992). Further, these days, so many of us interact with strangers regularly. Such interactions are actually relatively rare in small nomadic contexts. Finally, much of the communication that takes place in modern contexts is of the non-face-to-face variety—and, related, it often includes anonymous communication. These facts provide the evolution-based backdrop for the current study.

This work specifically focuses on a classic anti-social action that has been studied extensively in the field of evolutionary psychology: breaking a social contract (see Cosmides & Tooby, 1992). Primarily, this work sought to examine the effects of societal scale (small versus large) and contract communication type (via a written contract versus a handshake) in affecting how likely people are to break a contract.

### 1.1. The Evolutionary Psychology of Social Contracts

Social contracts are situations in which one individual must meet some requirement or pay some kind of cost to another individual to receive some kind of reward or benefit from said other individual (Cosmides, 1989). Cheating (in this context) involves taking the reward/benefit without paying the aforementioned cost or meeting the requirement (Kappesser & Williams, 2008). Therefore, for social contracts to evolve and be evolutionarily adaptive, we must have also developed some kind of cheater detection—a set of cognitive processes for detecting potential cheaters within a group (Kappesser & Williams, 2008).

Another key aspect of social contracts being evolutionarily adaptive pertains to punishment for cheaters who break their social contracts. Without this, cheating would always be the most adaptive thing to do when faced with a social contract, as cheaters will reap all of the benefits, while facing very minimal risk (Geher, 2014). Consequently, for social contracts to be adaptive, we do not just need a way of detecting cheaters—we also need a way to deter cheating from happening altogether. Thus, in ancestral times, those who were caught cheating were often ostracized from their group, which had potentially disastrous consequences for these individuals (see Geher et al., 2019a).

Thinking of social contracts at the group level, keeping such contracts intact maintains the network of implicit informal agreements and explicit formal agreements within a group of individuals (Raihani & Power, 2021). Such networks provide the baseline for the norms, attitudes, and shared beliefs within various relationships and are created through social promises, both formal and informal. That said, in large-scale societies that include many strangers along with anonymized communications, it may be the case that such networks are less cohesive and mutually supportive than would have been true of ancestral, small-scale human contexts.

The psychology of social contracts relies heavily on the evolutionary psychology of reputation, which focuses largely on a high premium placed on reciprocating prosocial acts (Geher et al., 2019b). Breaking social contracts can be a dangerous activity in small-scale groups where everyone knows one another. That said, in modern large-scale societies, the odds of being called out for breaking a social contract is diffused. And this fact, which follows from a major form of evolutionary mismatch, can be problematic in various ways. In short, such large-scale contexts seem to amplify anti-social behavior and downplay prosocial behavior (Figueredo et al., 2008).

### 1.2. Large- Versus Small-Scale Societies in Evolutionary Perspective

According to Dunbar’s number, 150 people is the maximum quantity of relationships that a person can cognitively handle (Lindenfors et al., 2021). This number is also approximately the average number of individuals that composed ancestral communities. For the bulk of human evolutionary history, people lived in small, tight-knit groups, made up mostly of our kin, creating an environment in which everyone knew each other very well. That said, today, 83% of the U.S population lives in urban environments (World Bank Group, 2025). In short, a large majority of people in the US (and in the world writ large; World Bank Group, 2025) live in urban, large-scale social contexts, acquiring, over time, a number of relationships that far surpasses 150.

In such large-scale contexts, people likely have less motivation to comply with contracts that are sealed with a handshake (or similar non-formal method) than was possibly the case under ancestral conditions. Thus, we can expect that people in urban contexts have less motivation to act in a prosocial manner—an expectation that is supported by various forms of data (see Figueredo et al., 2008). Critical evolutionary mismatches often bring adverse outcomes along for the ride (see Geher & Wedberg, 2022). In this case, it seems that living in large urban areas, which are inherently evolutionarily mismatched (i.e., containing relationship networks of greater than 150 people), seems to correspond to various kinds of anti-social behavior. In the current work, we see breaking a contract that is based on a handshake as an exemplar of such anti-social behavior. Importantly, it may also be the case that ingroup/outgroup processes (Billig & Tajfel, 1973; such as one from a small town overbenefiting someone if the framing is a small-town framing) may be at play. We elaborate on this possibility in the Discussion.

Our main prediction (further outlined later) is that framing certain messages about large or small scale living will influence participant’s self-reports of the likelihood that they would break a deal based on a written contract or a handshake—specifically, that when vignettes are framed in a small-scale societies, people will be more likely to keep a deal when it is made based on a handshake. This prediction is theoretically rooted in basic ideas in evolutionary social psychology (e.g., reciprocal altruism; Trivers, 1971).

On the heels of the work of Cosmides and Tooby (1992) and other work on social contracts that are steeped in the basic ideas of reciprocal altruism (Trivers, 1971), we propose a mechanism similar to that advanced by Cosmides and Tooby (1992). Namely, we propose that because of the high prevalence of reciprocal altruism in the broader human experience, humans evolved to essentially take steps to reciprocate prosocial acts in a highly discriminating manner. Further, we evolved to note the prosocial acts of others in a thoughtful, logical, and mathematically oriented manner. Toward this end, we evolved to conceptualize breaches of social contracts as particularly concerning as such breaches would signal that someone may take advantage of others for their own gain. And in a world where reciprocal altruism is so dominant, we evolved to carefully detect such cheating and make social decisions accordingly.

Toward this end, it is important to note that modern, large-scale conditions are highly mismatched from ancestral, small-scale conditions. In short, in large-scale conditions, we often encounter strangers whom we may never see again. Such a reality was not true under ancestral conditions and, as such, people did not evolve to be as prosocial toward strangers as they are toward kin and others whom they know well (see Zimbardo, 2007).

We propose that large-scale societies utilize formal mechanisms of sealing contracts (such as signed forms) to ensure behavioral control when people are dealing with strangers. Based on this reasoning, we predict that a large-scale social context, then, would lead people to follow rules related to formal, written contracts.

On the other hand, this same mechanism as related to societal scale interacting with our evolved social nature is predicted to bear on how people operate in small-scale social settings (that more closely match our evolved social psychology). We expect that when individuals are seeing a social context as small in scale, they would be less focused on the evolutionarily novel form of sealing a contract (signing a written form) relative to and more focused on evolutionarily old forms of sealing a contract that would necessarily have included face-to-face interaction (such as a handshake).

### 1.3. Dispositional and Demographic Factors Related to the Psychology of Contracts

There are several dispositional and demographic factors that might be related to people’s propensity to keep or break contracts, such as facets of the Dark Triad (Jonason & Webster, 2010), socioeconomic status, and area of upbringing.

For example, people high in dark triad traits—narcissism, Machiavellianism, and psychopathy—are more likely to take advantage of others (Jonason & Webster, 2010). As such, people who report being more self-focused and manipulative (i.e., who score highly in dark triad traits) are generally less likely to keep formal or informal contracts (Baran & Jonason, 2022). If someone scores highly on the dark triad, it is likely that the person will prioritize their own gain from the contract, rather than the benefit of the other party. If there is no risk except the loss of a social bond in breaking some hypothetical contract, an antisocial person should have no problem disregarding their end of the bargain. From past theoretical and empirical work, the varying traits composing the dark triad are so closely related that they tend to act as a unit (Jonason et al., 2013). As such, all traits within the dark triad are expected in the present study to closely relate with the propensity to break deals.

Socioeconomic status (SES) is also expected to relate to people’s propensity to report breaking deals, but the relationship between SES and unethical behavior is nuanced. Some research has shown that socioeconomic status is positively related to selfishness and unethical behavior—that is, richer people behave more selfishly (Piff et al., 2012).

For example, in laboratory studies, people of higher socioeconomic statuses were more likely than those with lower socioeconomic statuses to lie in negotiation and endorse unethical behavior at work (Piff et al., 2012). Nevertheless, members of both low and high socioeconomic status groups might behave unethically, but for different reasons (Dubois et al., 2015). Dubois et al. found that people of low socioeconomic statuses were more likely to engage in cheating/unethical behavior when the result of their unethical behavior was of benefit to other people, and people of higher socioeconomic statuses were more likely to engage in such behaviors when they themselves were going to benefit.

Given these nuanced relationships between socioeconomic status and unethical behavior, in the present study, we conducted an exploratory examination of the relationship between SES and the likelihood to break a deal.

Last, we predicted that people’s area of upbringing would map onto their reported likelihood to break a deal. In terms of the experimental element of this work, we expected that people would be more likely to break a deal sealed by a handshake when thinking about a rural environment and more likely to break a written contract when thinking about an urban environment. But in a more exploratory light, we also investigated whether being raised in an urban, suburban, or rural environment would relate to people’s likelihood to break deals, generally. In other words, we studied social context as both a manipulated independent variable in addition to studying this variable as a background (dispositional) variable (based on participants’ reports of the environments of their upbringing; see Section 2).

### 1.4. Primary Hypotheses

Using an evolutionary-mismatch-based conceptualization, this study examined the relationship between the scale of a social ecosystem (large versus small-scale) along with dispositional variables that were predicted to relate to the likelihood of breaking a deal. Our hypotheses were as follows:

**H1:**  *We predicted participants who were presented with a large-scale context to be relatively likely to break a deal based on a handshake.*

**H2:**  *We predicted participants who were presented with a small-scale context to be relatively likely to break a deal based on a written contract.*

**H3:**  *Across experimental conditions, we predicted participants who scored high on the Dark Triad to be relatively likely to break a deal.*

**H4:**  *Across experimental conditions, we predicted participants who reported growing up in a relatively urban context to be relatively likely to break a deal.*

**H5:**  *We had a non-directional hypothesis regarding the potential correlates of socioeconomic status as related to the likelihood of breaking a deal (in other words, we sought to see if self-reported SES was predictive of likelihood to break a deal in either direction).*

## 2. Method

For this research, adult participants completed an online survey that included an experimental manipulation. Participants were presented with one of four vignettes (representing two two-level independent variables: (a) large-scale versus small-scale context and (b) contract based on a handshake versus a written contract. The vignettes presented a scenario in which participants had agreed to purchase a house in a real estate deal but, based on new information, were presented with motivation to back out of the deal. We also measured several personality traits and demographic features of the participants to examine if some person features predicted the likelihood of breaking a deal in spite of experimental condition.

### 2.1. Participants

200 adult participants completed the survey for this study. These participants generally were young American adults (mean age = 19.60 (SD: 2.37); 132 reported being female; 48 reported being male; 13 reported being non-binary; 4 indicated that they preferred not to say; one did not answer this question. Several of the participants were recruited via the SUNY New Paltz Psychology Participant Pool. Others were contacted via social media and similar sources via a convenience-sampling process.

### 2.2. Materials

The survey, created via Qualtrics, included an experimental manipulation presenting participants with one of four vignettes that related to likelihood of breaking a deal (see Section 2.3 for more details on this process). In addition to being exposed to said vignette, participants were asked on a 1–5 Likert scale to report how likely they would be to break the deal.

Important demographic variables include gender identity, socioeconomic status of one’s upbringing (using the socioeconomic ladder measure, Adler et al., 2000), current socioeconomic status (using the same measure), along with three Likert-scale-based questions regarding the nature of the environment of their primary upbringing. Specifically, participants used a 1–5 scale to describe how rural, suburban, and urban the environment of their primary upbringing was.

Next, they also completed the Dirty Dozen (Jonason & Webster, 2010), which taps the three elements of the Dark Triad of personality via four items each (for narcissism [the tendency to over-value oneself], Machiavellianism [the tendency to manipulate others for one’s own gain], and psychopathy [the tendency to not have feelings for others]). This measure, with each item on a 1–7 Likert scale, was used to test the prediction that people who are high in the Dark Triad are more likely to break deals than are others—regardless of the experimental manipulation.

### 2.3. Procedure

Via random assignment to conditions (based on Qualtrics coding), participants were first presented with one of four vignettes. In each vignette, participants were provided a scenario in which they had made an agreement to purchase a house, but they were then provided information about a better house on the same street for a lower price that had just entered the market. Two independent variables were manipulated. First, participants were told either that their initial agreement was based on a handshake or on a signed written contract. Second, participants were told that this transaction was taking place in a very small town versus a very large city.

Participants then completed the demographic variables (described in Section 2). Data collection was completed fully through the Qualtrics environment.

## 3. Results

The main prediction of this work is interaction-based: We predicted that the type of environments participants envisioned—that is, small-town or big-city—would moderate participants’ decisions to either keep or break different types of contracts—namely, a handshake contract or a written contract.

Using a between-groups design, we specifically hypothesized the following: When thinking about a situation in which a deal was sealed with a handshake, participants envisioning a small-town context would be less likely to break the deal than those participants thinking about a large-city context. Conversely, we predicted that participants thinking about a large-city context would be less likely to break a written contract (as opposed to a handshake) than those thinking about a small-town environment. We used a two-way, between-groups ANOVA to see if these variables interacted in terms of their effects on the likelihood to break a contract.

We also collected various demographic variables that might bear on the proclivity to break a contract, such as age, socioeconomic status, and the community size of one’s place of upbringing. Similarly, we assessed various potentially relevant trait variables—most notably, the three facets of the Dark Triad (narcissism, Machiavellianism, and psychopathy). We used correlational and regression-based statistics to examine how these variables related to the likelihood to break a contract, regardless of experimental condition.

### 3.1. ANOVA Testing for Effects of Type of Agreement and Community Size

First, we conducted a 2 × 2 between-groups ANOVA to test the primary hypothesis, with community size (small town versus large city) as one independent variable and type of agreement (handshake versus signed, written contract) as the other independent variable. The dependent variable is the likelihood to break the deal (measured with a 1–5 Likert-scale item). The means and standard deviations for this analysis are found in Table 1.

**Figure 1:**
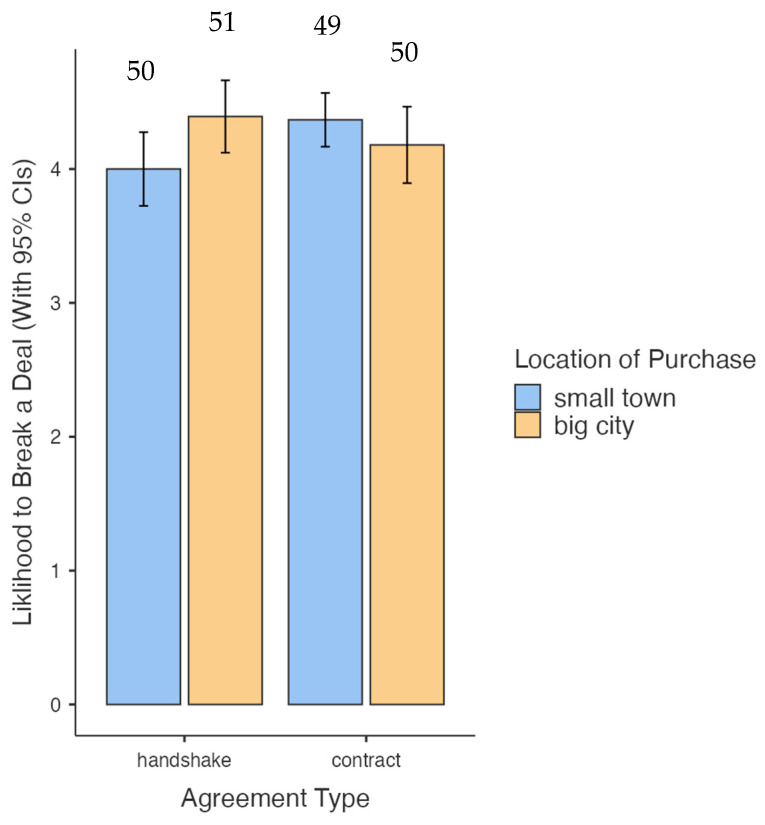
Interaction between Community Size and Type of Contract on Likelihood to Break a Deal (cell Ns are found above each bar); the likelihood to break a contract item was a 1–5 point Likert scale (with higher scores corresponding to being more likely to break a deal).

Interestingly, neither of the two independent variables had main significant effects on the dependent variable (Type of Agreement: *F*(1, 196) = 0.358, *ns*); Community Size: *F*(1, 196) = 0.623, *ns*). However, the interaction between these two independent variables (see Figure 1) was significant (*F*(1, 196) = 4.99, *p* < 0.05, partial *η*^2^ = 0.025).

In other words, neither thinking of a city nor thinking of a small town had an independent effect on one’s likelihood of breaking a deal. The community size about which one thought, on the other hand, moderated the likelihood of breaking a deal based on a handshake or written contract. This effect was driven by the fact that, in the small-town condition, participants were more likely to break a deal if the deal was solidified by a written contract (and less likely to break a deal sealed by a handshake). In the large-city condition, the opposite effect was found: Participants who thought about a large city were more likely to break a deal if it was made by a handshake, and they were less likely to break a deal sealed by a written contract.

Recall that participants were largely recruited via an ad hoc sampling process, so the effect should be interpreted accordingly.

### 3.2. Correlation and Multiple Regression Testing: Demographic and Trait Variables

Descriptive statistics for the primary variables are provided in Table 2. As can be seen therein, the sample was constituted by 200 young adults (mean age was 19.60, SD = 2.37). They were mostly drawn from the SUNY New Paltz Psychology Department Participant Pool.

Predicting the likelihood to break a deal was the primary outcome measure in this study. The next set of analyses aimed to examine the zero-order correlations between the primary predictor variables with this critical outcome measure (likelihood to break a deal; see Table 3). Of these correlations, two were statistically significant. Specifically, these were the positive correlation between likelihood to break a deal and the degree to which one’s upbringing was primarily urban (*r*(188) = 0.25, *p* < 0.01) and the negative correlation with childhood SES (*r*(183) = −0.16, *p* < 0.05).

To examine the overall amount of variability in the likelihood to break a deal explained by area of upbringing and socioeconomic status, and to further explore the amount of variability each variable contributes to this likelihood, a multiple regression analysis was conducted (see Table 4). The analysis aimed to capture how these predictors, in combination, relate to the criterion variable. The results indicated that the set comprising area of upbringing and socioeconomic status accounted for a significant portion of the variability in the likelihood to break a deal (*R*^2^ = 0.09, *F*(4,163) = 3.94, *p* < 0.01), signifying that approximately 9% of the variability could be explained by this combination of factors.

Further analysis through semi-squared partial correlations was utilized to ascertain the unique contributions of area of upbringing and childhood socioeconomic status. Specifically, we examined the degree to which participants’ areas of upbringing were urban, suburban, or rural in their relation to likelihood to break a deal. The findings were revealing: Growing up in an urban area significantly affected the likelihood to break a deal (*sr*^2^ = 0.07, *p* < 0.001), suggesting that approximately 7% of the variance in likelihood to break a deal can be accounted for by growing up in an urban area. Similarly, growing up in a rural area also accounted for a small portion of the variance in likelihood to break a deal (*sr*^2^ = 0.03, *p* < 0.05). Conversely, growing up in a suburb did not account for a significant increase in likelihood to break a deal (*sr*^2^ = 0.02, *ns*) and the same was the case for socioeconomic status (*sr*^2^ = 0.00, ns). Interestingly, scoring highly on the elements of the Dark Triad seemed to have no utility in predicting likelihood to break a deal.

## 4. Discussion

Based on the basic idea of evolutionary mismatch in our modern social ecologies (relative to the small-scale social ecologies that were most prevalent during human evolutionary history), this study sought to examine the size of a community along with the nature of how an agreement was made would have effects on whether an individual would break a social contract. The main prediction, which was supported, was interaction-based. We predicted that people presented with large-scale contexts would be less likely to break a deal if that deal was based on a written contract, while we predicted that people presented with small-scale contexts would be less likely to break a deal if said deal was based on a handshake.

We also predicted that participants who scored as having Dark Triad personality traits would be more likely to break a deal across the board. Further, we expected that people with relatively little financial means (scoring low on our indices of socioeconomic status) would vary from people with more financial means in terms of likelihood to break a deal (direction of relationship was not specified). We were also curious if the environment of one’s upbringing (rural, suburban, urban) would predict the likelihood to break a deal (e.g., perhaps people from large-scale, urban areas would be more likely to break a deal as their developmental background prepared them for relatively large-scale, evolutionarily mismatched living).

### 4.1. The Effects of Social Scale and of Nature of Contract on Probability of Breaking the Contract

As shown in Figure 1, participants framed with the small-town condition were more likely to break the contract when their initial agreement was based on a written contract, as opposed to a handshake. On the other hand, participants framed with the large city condition were more likely to break the contract when their initial agreement was based on a handshake, as opposed to a written contract. Thus, our primary hypothesis was supported. This finding dovetails with work in the field of evolutionary psychology (e.g., Dunbar, 1992) in that small-scale living is, by definition, better-matched to our ancestral eco-systems relative to large-scale living.

Though what we consider to be “small towns” in modern society often contain more than 150 people, those communities still mimic Dunbar’s Number in a relative sense. In a small town, it is not feasible or beneficial to break a contract sealed by handshake, due to the potential threat that the breach of trust poses to one’s reputation and the overall sense of social cohesion within the community. Showing a disregard for others by breaking a handshake-formed contract in such a context could have damaging reputational effects (see Geher et al., 2019a). However, if an individual from a big city were to break a contract sealed by handshake, the potential social ramifications would be much-diffused.

This analysis is consistent with the evolutionary mismatch framework that surrounds this study: Under large-scale societal conditions, people are relatively deindividuated and anonymous. This context seems to breed anti-social actions (see Figueredo et al., 2008)—such as breaking a deal that was sealed with a handshake.

### 4.2. Dispositional and Demographic Correlates of Breaking Deals

Our correlational analyses show that urban upbringing and socioeconomic status in childhood were significant predictor variables for the likelihood to break a deal in a zero-order correlation. Our more fine-tuned multiple regression analysis, on the other hand, found only urban upbringing and rural upbringing to be significant predictors of the likelihood of breaking a deal, while socioeconomic upbringing was not. Lastly, Dark Triad traits had no significant influence on the outcome measure.

These results can be understood in light of game theory and anonymity in large groups (Axelrod, 1984). When making decisions, people must weigh the costs and benefits of their actions. In some cases of cooperation, it may be strategic for an individual to betray others in order to benefit more from doing so, even if the group suffers as a result (Vlerick, 2020). However, when living in small, stable social groups, the cost of betraying someone can be detrimental.

Acts of betrayal can lead to punishment, exile, and even revenge (Spikins, 2015; Fitness, 2001; Ruel et al., 2022; Combs et al., 2010). For this reason, it may be advantageous to avoid breaking contracts in small groups, such as in rural settings, to preserve one’s reputation. However, the cost–benefit analysis may differ in large groups, such as cities. In urban settings, there is less concern for public reputation compared to rural settings (Danielson et al., 2023). A factor in this decreased concern for reputation may be that it is much easier to keep one’s personal life private in cities than in rural communities (Haugen & Villa, 2006; Leung et al., 2015). With greater anonymity, a person can get the same level of benefits from betraying someone without risking the victim seeking revenge. A betrayer might even be able to maintain benefits from past relationships when breaking a deal under the anonymity of a city.

Victims of private acts of betrayal are more likely to help the betrayer in the future than if it were a public act of betrayal (Ruel et al., 2022; Combs et al., 2010). In an urban setting, an individual also might not have to worry about how they will be perceived after breaking a deal, since most people they meet may not be aware of their past interactions. On the other hand, individuals in a rural setting may find it harder to conceal their history of breaking a deal.

Socioeconomic upbringing also appears to have a potential influence on one’s likelihood of breaking a deal—at least based on the zero-order correlations. Here, we found a small effect for people who grew up as relatively poor to be more likely to break the deal. This outcome can be understood by examining risk-aversion and Life History Strategy. Individuals from lower socioeconomic backgrounds tend to be more risk-averse, especially when dealing with already scarce resources (Hilbert et al., 2024; Guiso et al., 2013; Weissberger et al., 2022; Grable, 2000). People from low-SES backgrounds are also more likely to have a fast life history strategy, prioritizing a short-term focus on reproduction and survival. For this reason, people from low SES in childhood might be more likely to prioritize immediate financial benefits over long-term relationships (Figueredo et al., 2014; Figueredo et al., 2007).

Lastly, there was a surprising finding for the Dark Triad. The Dark Triad traits actually had little effect on the likelihood of breaking a contract. Some correlation was found, where an increase in these traits also increased the likelihood to break a contract. However, the influence was not significant enough for the Dark Triad to be a reliable predictor for someone’s chances of breaking a deal. In other words, our prediction about the close relationship between the propensity to break a deal and being high in Dark Triad traits did not emerge as significant in the current study.

### 4.3. Limitations of Current Research and Implications for Future Research

As is the case with any study, this work has its limitations. First, as is often a problem in psychological research, the questions asked are primarily of the self-report variety. While seeing if people are likely to report that they think that they would break a deal in some context is interesting, it may not align with what people might actually do in such a scenario. A study that replicates this work conceptually but that presents real people who are in the market for a house with realistic in vivo scenarios might be useful to see if these findings replicate using more ecologically valid methods.

Further, the participant pool in the current study is a bit limited. Most young adults have likely not ever purchased real estate. It might be useful in future research to examine if these same effects are found for people who have made some number of real estate deals in their time.

Additionally, this work focuses on a particular kind of contract-breaking—it specifically focuses on going back on a real estate deal. In life, in fact, there are many forms of breaking a contract (see Cosmides & Tooby, 1992). People may break contracts in intimate relationships; they may break contracts when it comes to agreements among friends; people may break contracts in the workplace, etc. Future work could benefit from exploring these different posited avenues of research based on the limitations of the current study.

While the random assignment to conditions makes it very likely to be the case that people across all kinds of background variables (e.g., area of upbringing) are essentially equally distributed across groups, an important alternative hypothesis exists regarding our primary findings. Namely, ingroup/outgroup reasoning (see Billig & Tajfel, 1973) stands as a potential alternative explanation. While our main explanation focuses on the idea that we evolved for small-scale living and, thus, are more accepting of a handshake in sealing a deal relative to a written agreement when stimuli are framed in terms of small-scale societal features (and vice versa for when stimuli are framed in terms of large-scale societal features), the manipulation that we used may also elicit ingroup/outgroup reasoning. For instance, if someone is from a small town and is randomly assigned to the small-town condition, that person may be accepting of a handshake for sealing a deal based on the evolutionary reasoning presented above, or alternatively, that person may be likely to accept a handshake in a small-scale context as they are essentially behaving in a prosocial manner to someone with similar characteristics as themself.

Future research could be done to utilize participant background as a between-participants factor (small-scale versus large-scale upbringing) and adding this variable into this methodological paradigm would allow for the assessment of whether an ingroup/outgroup effect is at work in shaping the results. It may be that the ingroup/outgroup manipulation has no effect or a partial effect. Or it may be that the ingroup/outgroup manipulation explains the outcomes fully. This is an important and provocative alternative hypothesis that should be examined carefully in future research.

One limitation of this work pertains to the issue of ecological validity. The main concern here is that most college students likely have never purchased real estate. Our initial justification for using this scenario partly stems from the work of Cosmides and Tooby (1992) who provided stimuli across a broad array of social spheres. Some were highly familiar to US college students (e.g., taking a cab versus a train) and some were intentionally highly unfamiliar (e.g., making a judgment about the actions of a leader of a hypothetical nomadic group in Polynesia). Their research found that the social-contract relevance ended up affecting judgments the same regardless of whether the contents were highly familiar or not. With this in mind, it may be reasonable to infer that the house-purchase vignette used in the current study, while perhaps not relevant to the lived experiences of many young adults, would have facilitated social contract-reasoning appropriately.

This said, there is an issue of ecological validity that emerges. It would be interesting to see whether, in using our methodological paradigm, more age-appropriate scenarios would have similar effects. For instance, many students sign leases for apartments for off-campus living. Perhaps scenarios that manipulated whether agreements regarding leasing were made via handshakes or signed contracts would have been more age-appropriate. In future research, it would be very interesting to see if outcomes with a more age-appropriate (and perhaps ecologically valid) set of stimuli would have been similar to the outcomes that we observed in the current study.

### 4.4. Implications for Living

The findings presented here have various implications regarding our social worlds. In terms of the experimental manipulation, we found that people presented with a large-scale social context are less likely to keep a deal based on a handshake, while, conversely, people presented with a small-scale social context are less likely to keep a deal based on a written, signed contract. These findings have important implications for conducting business with others. If one is conducting business in a small town among friends or other familiar people, it might be useful to realize the value of a handshake in such contexts. If one is doing business in a large-scale social context, it seems that getting agreements in writing seems particularly important.

Area of upbringing had significant effects on the likelihood of breaking a deal—and these effects cut across experimental conditions. In short, people who reported having been raised primarily in urban areas were significantly more likely to report that they would break a deal relative to others. This finding is very much in line with the primary mismatch-based focus of the current work.

Further, a small (yet still significant) effect was found such that people who reported having grown up in relatively rural areas were also more likely to report that they would break a deal. While this effect was considerably smaller than was the effect for an urban upbringing, it deserves attention as it is incongruous with our basic prediction and, as such, warrants further study.

In any case, via utilizing the evolutionary mismatch approach that underlies the current work, it may be the case that the scale-of-area of one’s upbringing will demonstrate to be predictive of a broad suite of social-relevant outcome measures in future research.

## 5. Conclusions

The evolutionary mismatch concept has demonstrated a great deal of heuristic value in the evolutionary behavioral sciences (see Giphart & Van Vugt, 2018). In the modern industrial age, mismatches abound. And they bear on so many features of our worlds, including physical health (O’Keefe et al., 2006), education (Gruskin et al., 2025), politics (Geher et al., 2015), and social ecology (see Wilson, 2019).

One of the core mismatches that surrounds our modern worlds pertains to group-size. Under ancestral conditions, nomadic human clans rarely exceeded 150 (see Dunbar, 1992). Under such conditions, strangers were pretty much non-existent; people were surrounded by others whom they would interact with day in and day out, likely for the rest of their lives. Further, breaking a contract with someone in one’s clan could have had damaging effects to one’s own reputation and ultimately to one’s own capacity for survival and reproduction. Further, during this era of human evolutionary history, communication never included written contracts and signatures. Processes such as a simple handshake were, when it came to sealing an agreement, pretty much the only game in town.

In large-scale social contexts, people seem to respect a handshake agreement less than is the case in a small-scale social context. This information, along with the evolutionary mismatch concepts that surround it, might help people create contexts for forming agreements with others that better match our evolved psychology—an outcome that may well push the needle of harmony among people in a positive direction.

## Figures and Tables

**Table 1:** Means and Standard Deviations across Levels of Community Size and Type of Agreement (with Likelihood to Break a Deal as the Dependent Variable).

Agreement Type	Location of Purchase	Likelihood of Breaking Deal (1–5) Mean	SD	N
Handshake	Small town	4	0.97	50
	Big city	4.39	0.96	51
	Total	4.19	0.98	101
Contract	Small town	4.37	0.7	49
	Big city	4.18	1	50
	Total	4.27	0.87	99
Total	Small town	4.18	0.86	99
	Big city	4.29	0.98	101
	Total	4.24	0.92	200

**Table 2:** Descriptive Statistics for Continuous and Categorical Primary Predictor Variables.

**Continuous Predictor Variables**	**N**	**Mean (SD)**
Age	187	19.60 (2.37)
Ladder (Childh) ^1^	183	4.98 (1.74)
Dirty Dozen (total): ^2^		
Machiavellianism	199	11.03 (5.04)
Psychopathy	197	7.40 (3.89)
Narcissism	194	13.26 (5.27)
PL_Urban ^3^	188	2.80 (1.44)
PL_Suburban	189	3.65 (1.38)
PL_Rural	182	2.13 (1.34)
**Categorical Predictor Variables**	**N**	**%**
Gender Iden.	199	
Female	132	66.33
Male	48	24.12
Non-binary	13	6.53
PNS *	4	2.01

* Key: ^1^ Ladder (Childh) = Where was your family on this ladder during your childhood? (1–10 point Likert scale); ^2^ the three Dirty Dozen subscales all comprised four 5-point Likert scale items; ^3^ these three scales all used a 1–5 Likert scale format. PL = Primary Location of Upbringing; PNS = Prefer not to Say.

**Table 3:** Zero-Order Correlations among Key Predictor Variables and Likelihood to Break a Deal.

	Age	PLU Urban	PLU Sub.	PLU Rural	SES Child-Hood	Machiavellianism	Psychopathy	Narcissism
Break Deal	0.011	0.246 **	−0.092	0.039	−0.161 *	0.088	0.026	0.024
N	187	188	189	182	183	199	197	194

** Correlation is significant at the 0.01 level (2-tailed). * Correlation is significant at the 0.05 level (2-tailed). PLU: Place of Upbringing.

**Table 4:** Multiple Regression Predicting Likelihood to Break a Deal from Key Predictor Variables.

Key Predictor Variables	*b*	*B*	*sr* ^2^
Urban Area of Upbringing	0.24	0.38	0.07 **
Suburban Area of Upbringing	0.10	0.16	0.02
Rural Area of Upbringing	0.15	0.22	0.03 *
SES-Childhood	−0.03	−0.06	0.00

R^2^ = 0.09, F(4,163) = 3.94, *p* < 0.01. ** Correlation is significant at the 0.01 level (2-tailed). * Correlation is significant at the 0.05 level (2-tailed).

## Data Availability

The original data presented in the study are openly available in Open Science Foundation (OSF) at: DOI:10.17605/OSF.IO/RZJEN.
